# Impacts of stroke and cognitive impairment on activities of daily living in the Taiwan longitudinal study on aging

**DOI:** 10.1038/s41598-021-91838-4

**Published:** 2021-06-09

**Authors:** Pi-Hsia Lee, Ting-Ting Yeh, Hsin-Yen Yen, Wan-Ling Hsu, Valeria Jia-Yi Chiu, Shu-Chun Lee

**Affiliations:** 1 School of Nursing, College of Nursing, Taipei Medical University, Taipei, Taiwan; 2 Master Degree Program in Healthcare Industry, Chang Gung University, Taoyuan, Taiwan; 3 School of Gerontology Health Management, College of Nursing, Taipei Medical University, Taipei, Taiwan; 4 Department of Rehabilitation Medicine, Taipei Tzu Chi Hospital, Buddhist Tzu Chi Medical Foundation, New Taipei, Taiwan

**Keywords:** Geriatrics, Quality of life, Dementia, Stroke

## Abstract

Stroke and cognitive impairment are common in older population. They often occur together and their combined effects significantly increase disability in both basic (BADLs) and instrumental (IADLs) activities of daily living. We investigated the individual and combined impacts of stroke and cognitive impairment on BADLs and IADLs. A total of 3331 community-dwelling older adults were enrolled from the Taiwan longitudinal study on aging in 2011. Both BADLs and IADLs were analyzed. Combination of stroke and cognitive impairment increased severity of ADL disabilities, but similar prevalence, similar numbers of summed BADL and IADL tasks with disability, and similar levels of difficulty for each BADL and IADL task were found between the stroke group and cognitive impairment group. The former had more difficult in dressing while the latter had more difficult in using the telephone, transport, and managing finances. A hierarchy of ADLs was also observed in all groups. ADL skill training supplemented with cognitive and physical interventions should focus on secondary prevention of dementia and improve motor functional capacity to reduce loss of ADLs.

## Introduction

Approximately 70% of stroke patients have cognitive impairment in the first year after the stroke^1, 2^. The prevalence at 3 years post-stroke ranges from 50 to 58%^3^, indicating that the prevalence of cognitive impairment after a stroke is very high and may progress to dementia. On the other hand, cognitive decline is also a risk factor of stroke. Lower cognitive function is associated with a higher risk of incident stroke^4^. There seems to be a reciprocal relationship between stroke and cognitive impairment. Both stroke and cognitive impairment impact cognitive and physical functions and contributes to disability^5, 6^. They interact resulting in disability that is greater than the sum of the independent contributions from either stroke or cognitive impairment alone^7^.

Basic activities of daily living (ADLs; BADLs) refer to the basic tasks of living that is an indicator of a person’s functional status, while instrumental activities of daily living (IADLs) address a higher level of ADLs that consist of more complex daily tasks that must be performed to continue living independently in the community^8^. Individuals with ADL disabilities have poor life satisfaction^9^, quality of life^10^, and find it difficult to return to the community.

Previous studies showed that 40% of acute stroke survivors had total or severe ADL disabilities at hospital discharge^11^. Nearly 40% of patients at 6 years post-stroke still had dependence in BADLs^12^. Stroke patients who had the worse ability to perform IADL tasks tended to have a poorer walking capacity, upper limb strength, and neurological recovery^11^. The majority of chronic stroke patients need assistance with bathing, and half of them cease such activities as preparing meals, doing housework, shopping, and walking outdoors^13^. As for dementia patients, around 40% of them are unable to perform any of the IADL tasks^14^. IADLs are usually impaired in the early stage of cognitive decline, but BADLs are affected later in the course of the condition^15^. The proportions of those independent in BADL tasks among mildly, moderately, and severely demented patients were 63%, 36%, and 0%, respectively^16^. Deficits in ADLs were correlated with memory, psychomotor speed^17, 18^, and executive functions^19^.

The majority of previous studies examined the effects of either stroke or cognitive impairment on ADL performances, but limited research has investigated the combined effects of both, although many studies have reported that cognitive impairments after a stroke can aggravate ADL limitations^20, 21^. Only one population study analyzed the combined effects of stroke and dementia on ADLs, and the results showed that individuals with stroke and dementia comorbidity had 1.8 out of 7 ADL limitations, compared to 1.4 for those with dementia, 0.6 for those with a stroke, and 0.3 for healthy controls without further analysis or comparison^7^. Therefore, using data obtained from a national representative sample, we aimed to investigate the individual and combined impacts of stroke and cognitive impairment on both BADLs and IADLs.

## Results

### Baseline characteristics

The original sample comprised 3727 people, 396 people with incomplete SPMSQ data were excluded, and therefore 3331 adults were included in the current study. There were significant differences among the control, stroke, cognitive impairment, and combination groups in all baseline characteristics including sex, educational level, age, marital status, numbers of comorbidities, cognitive functions, and upper and lower limb functions (all *p* < 0.001) (Table 1). About 3.5% of the participants were stroke survivors, 11.7% had cognitive impairments, and 1.8% had both conditions. Compared to similar ratios of males to females in the control and combination groups, greater proportions were male in the stroke group and female in the cognitive impairment group. More than 90% of individuals in the cognitive impairment group had lower educational attainment, and they were generally older and living without partners. The stroke and combination groups had more comorbidities. Half of the individuals in the cognitive impairment and combination groups had only mild cognitive decline. Compared to the worst limb functions in the combination group, a slightly larger proportion of individuals in the stroke group had poorer upper limb function, while those in the cognitive impairment group had poorer lower limb function.

**Table 1:** Demographic data, comorbidities, cognitive and physical function of study participants.

	Total	Controls		Stroke		Cognitive impairment		Combination		X^2^	*p* value
	*N*	*n*	%	*n*	%	*n*	%	*n*	%		
**Sample size**	3331	2763	82.9	118	3.5	391	11.7	59	1.8		
**Sex**											
Male	1644	1444	52.3	77	65.3	98	25.1	25	42.4	114.73	< 0.001
Female	1687	1319	47.7	41	34.7	293	74.9	34	57.6		
**Education**											
Elementary school	2221	1726	62.5	75	63.6	367	93.9	53	89.8	169.42	< 0.001
Junior high school	373	344	12.5	16	13.6	12	3.1	1	1.7		
Senior high school	553	516	18.7	22	18.6	10	2.6	5	8.5		
College and above	187	177	6.4	5	4.2	2	0.5	0	0.0		
**Age (years)**											
55–65	1276	1220	44.2	27	22.9	23	5.9	6	10.2	436.93	< 0.001
65–75	1010	851	30.8	46	39.0	94	24.0	19	32.2		
75–85	768	535	19.4	32	27.1	173	44.2	28	47.5		
85+	277	157	5.7	13	11.0	101	25.8	6	10.2		
**Marital status**											
Married	1089	769	27.8	34	28.8	253	64.7	33	55.9	227.06	< 0.001
Single	2242	1994	72.2	84	71.2	138	35.3	26	44.1		
**No. of comorbidities (0–7)**											
0–2	2734	2307	83.5	72	61.0	321	82.1	34	57.6	63.34	< 0.001
≥ 3	597	456	16.5	46	39.0	70	17.9	25	42.4		
**Cognitive function**											
Normal	2881	2763	100.0	118	100.0	0	0.0	0	0.0	3332.00	< 0.001
Mild impairment	254	0	0.0	0	0.0	222	56.8	32	54.2		
Moderate impairment	167	0	0.0	0	0.0	144	36.8	23	39.0		
Severe impairment	29	0	0.0	0	0.0	25	6.4	4	6.8		
**Upper limb function**											
No difficulty	3182	2698	97.6	96	81.4	351	90.2	37	62.7	341.769	< 0.001
Some difficulty	92	51	1.8	12	10.2	22	5.7	7	11.9		
Great difficulty	36	11	0.4	7	5.9	10	2.6	8	13.6		
Inability	21	5	0.2	3	2.5	6	1.5	7	11.9		
**Lower limb function**											
No difficulty	2798	2482	89.9	76	64.4	222	57.1	18	30.5	508.164	< 0.001
Some difficulty	159	104	3.8	4	3.4	43	11.1	8	13.6		
Great difficulty	127	79	2.9	9	7.6	33	8.5	6	10.2		
Inability	247	100	3.6	29	24.6	91	23.4	27	45.8		

### Prevalence of disabilities among groups

Prevalence of disabilities in BADLs and IADLs significantly differed among the groups (X^2^ = 690.985, *p* < 0.001). More than 60% of individuals in the combination group had both BADL and IADL disabilities, but only 30% of individuals in either the stroke or cognitive impairment groups had both kinds of disabilities. The proportion of individuals in the control group who had disabilities in both BADLs and IADLs was only 5% (Table 2). The hierarchical relationship between IADLs and BADLs was observed in all groups, in which IADLs were first affected, followed by both IADLs and BADLs.

**Table 2:** Prevalences of disabilities in basic activities of daily living (BADLs) and instrumental ADLs (IADLs) among groups.

Group	Controls		Stroke		Cognitive impairment		Combination	
	*n*	%	*n*	%	*n*	%	*n*	%
No disability	2090	75.6	51	43.2	110	28.1	5	8.5
IADL disability only	541	19.6	32	27.1	180	46.0	17	28.8
BADL disability only	5	0.2	0	0	0	0	0	0
Disabilities in both BADLs and IADLs	127	4.6	35	29.7	101	25.8	37	62.7

### BADL disabilities among groups

**Figure 1:**
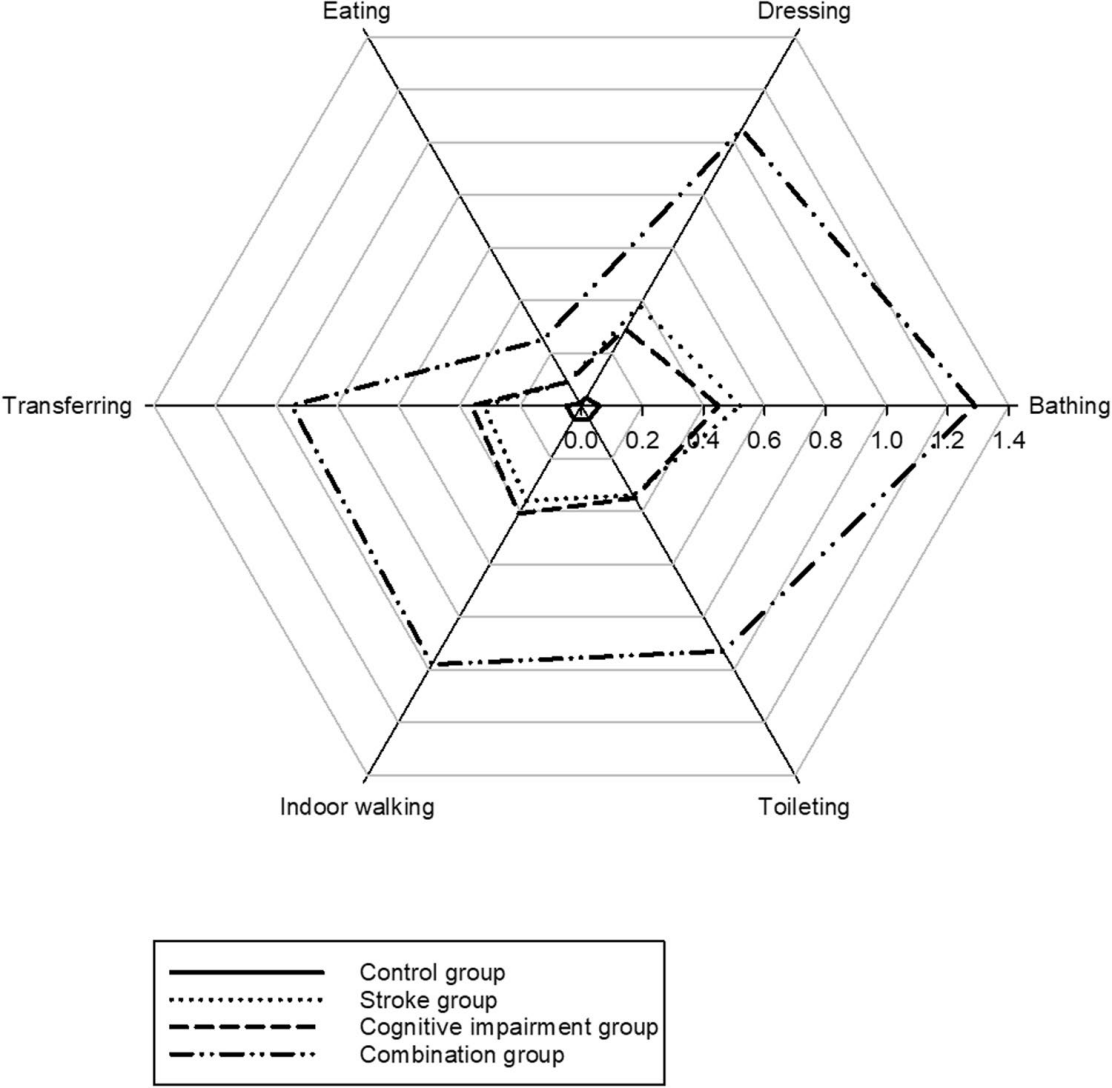
The level of difficulty for each basic activity of daily living (BADL) task presentated as mean values among the control, stroke, cognitive impairment, and combination groups. (0): no difficulty; (1): some difficulty; (2): great difficulty; and (3): inability to perform the task.

Among the six BADL tasks, the number of disabilities of BADL tasks was significantly different among groups (F(3,3322) = 126.836, *p* < 0.001). The post-hoc analysis showed that the combination group had the greatest number of disabilities of BADL tasks (2.56 ± 0.73) compared to the cognitive impairment (0.95 ± 1.88, *p* < 0.001), stroke (1.04 ± 0.73, *p* < 0.001), and control groups (0.14 ± 0.73, *p* < 0.001), but there was no difference between the stroke and cognitive impairment groups (*p* = 0.296). Moreover, the level of difficulty in all BADL tasks was significantly different among groups (F(3,3322) = 115.998, *p* < 0.001 in bathing, F(3,3322) = 110.196, *p* < 0.001 in dressing, F(3,3322) = 54.061, *p* < 0.001 in eating, F(3,3322) = 89.260, *p* < 0.001 in transferring, F(3,3322) = 81.289, *p* < 0.001 in indoor walking, and F(3,3322) = 86.873, *p* < 0.001 in toileting). The combination group had the greatest difficulty in all BADL tasks than all of the other groups (all *p* < 0.001). No differences were found again in the majority of BADL tasks between the stroke and cognitive impairment groups, but the stroke group had a significantly greater difficulty than the cognitive impairment group in dressing (*p* = 0.04) (Fig. 1). Regardless of the group, the most difficult BADL task was bathing, while the least difficult task was eating.

### IADL disabilities among groups

**Figure 2:**
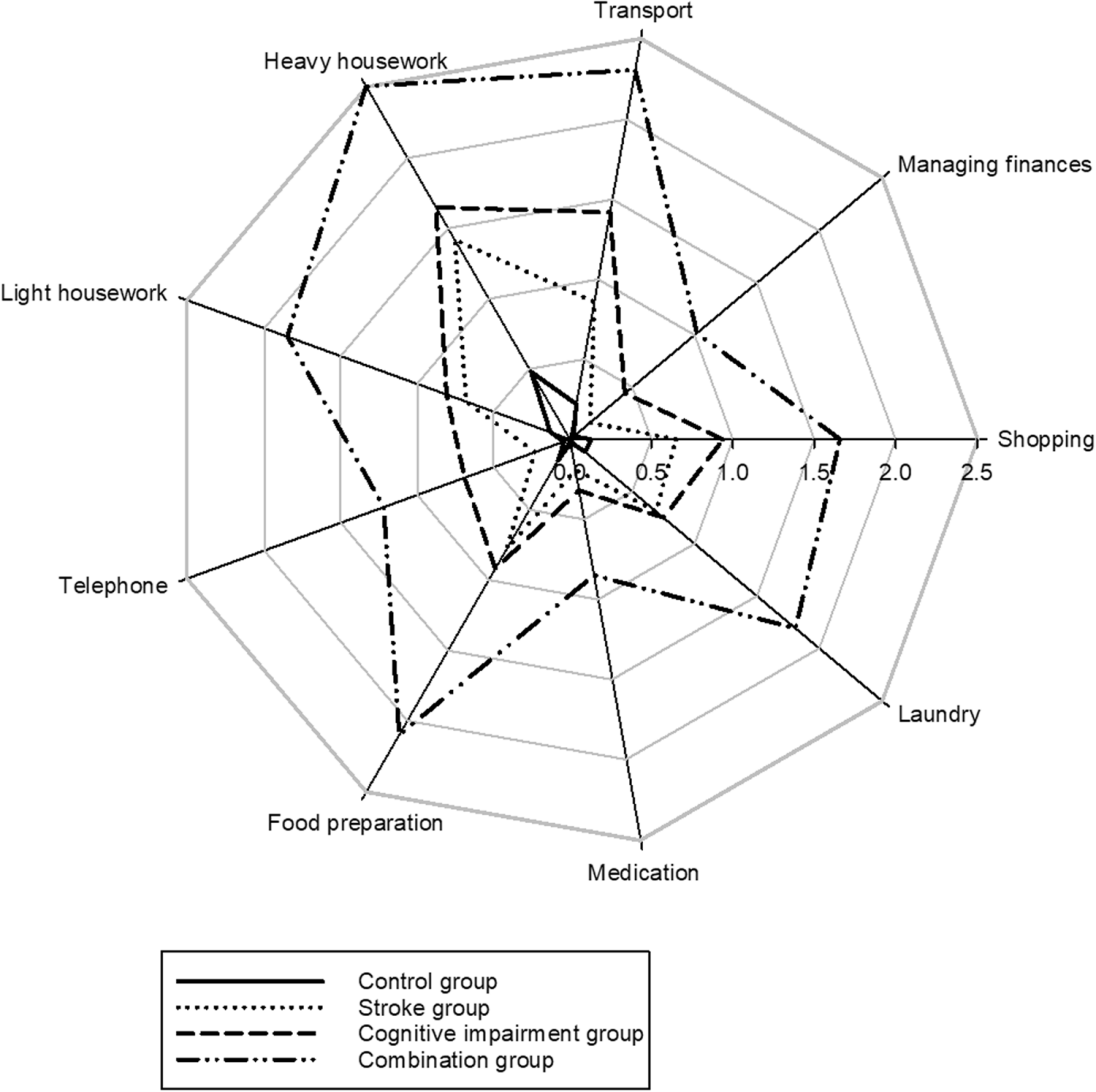
The level of difficulty for each instrumental activity of daily living (IADL) task presentated as mean values among the control, stroke, cognitive impairment, and combination groups. (0): no difficulty; (1): some difficulty; (2): great difficulty; and (3): inability to perform the task.

Among the nine IADL tasks, the number of disabilities of IADL tasks was significantly different among groups (F(3,3322) = 230.389, *p* < 0.001). The post-hoc analysis showed that the combination group had the greatest number of IADL tasks with disabilities (6.00 ± 3.05) compared to the cognitive impairment (3.25 ± 3.11, *p* < 0.001), stroke (2.34 ± 3.11, *p* < 0.001), and control groups (0.61 ± 1.51, *p* < 0.001), but there was no difference between the stroke and cognitive impairment groups (*p* = 0.195). Furthermore, the level of difficulty in all IADL tasks was significantly different among groups (F(3,3322) = 110.229, *p* < 0.001 in light house work, F(3,3322) = 58.100, *p* < 0.001 in heavy housework, F(3,3322) = 156.482, *p* < 0.001 in transport, F(3,3322) = 131.282, *p* < 0.001 in managing finances, F(3,3322) = 106.933, *p* < 0.001 in shopping, F(3,3322) = 129.330, *p* < 0.001 in laundry, F(3,3322) = 133.894, *p* < 0.001 in medication, F(3,3322) = 133.155, *p* < 0.001 in food preparation, and F(3,3322) = 194.000, *p* < 0.001 in using the telephone). The combination group had the greatest difficulty in all IADL tasks compared to the other groups (all *p* < 0.001). No differences were found in the majority of IADL tasks between the stroke and cognitive impairment groups, but the cognitive impairment group had a significantly greater difficulty than the stroke group in using the telephone (*p* < 0.001), transport (*p* = 0.002), and managing finances (*p* < 0.001) (Fig. 2). Regardless of the group, the most difficult IADL task was heavy housework, while the least difficult was taking medications.

## Discussion

In this nationally representative sample of middle-aged and older community-dwelling adults, we found that individuals with a combination of stroke and cognitive impairment had the highest prevalence of disabilities in both BADLs and IADLs, while individuals with either stroke or cognitive impairment had the lowest prevalence. Similar prevalences were found in the stroke and cognitive impairment groups. Individuals with a combination of stroke and cognitive impairment had the greatest number of total BADL and IADL tasks with disability compared to those with either of these conditions alone. The level of difficulty in all tasks experienced by individuals with stroke or cognitive impairment was less than the sum of the combined contributions from stroke and cognitive impairment. However, in most cases there were no differences between the stroke and cognitive impairment groups, but the former had more difficulty with a BADL task (dressing) and the latter had more difficulty in some IADL tasks (using the telephone, transport, and managing finances). Regardless of the group, the most difficult BADL and IADL tasks were respectively bathing and heavy housework, while the least difficult ones were eating and taking medication.

The prevalence of disabilities in people with combined stroke and cognitive impairment was higher than those with either of these conditions alone. Previous studies concluded that stroke patients with cognitive impairments having worse ADL disabilities than those without^20, 21^. However, the prevalence in either the stroke or cognitive impairment group in the current study were relatively lower than those in previous studies^12, 16, 22^. This could be because individuals in the present study were community-dwelling adults, who may have had better functional statuses and independent abilities with better cognitive and physical functions than individuals living in the institutions. Another interesting finding was the hierarchical relationship between IADL and BADL disabilities that existed in all groups. Prior research reported the sequential relationship of the majority of older adults having mobility limitations first and then IADLs being affected, and finally BADLs being impaired^23^. Hoeymans and colleagues further categorized disability into four levels-no disability, IADL disabilities only, combination of IADL disabilities and mobility limitations, and both IADL and BADL disabilities^24, 25^. Yeh and colleagues further determined cutoff points of dependence with IADLs and BADLs when individuals had difficulties with four and seven of nine items, respectively, in the physical mobility domain^23^.

Individuals with combined stroke and cognitive impairment had the greatest number of summed BADL and IADL tasks with disabilities compared those with either of these conditions alone, which is in line with previous studies^7^. The current study additionally analyzed the level of difficulty with each IADL and BADL task among groups, and unsurprisingly showed that the combination group had the highest levels of difficulty compared to all other groups. It is worth noting that no differences were found in the majority of BADL and IADL tasks between the stroke and cognitive impairment groups, but the stroke group had more difficulty than the cognitive impairment group with dressing. Dressing requires upper limb function, which was reported to be more associated with ADLs, while lower limb function is more related to IADLs^26^. The stroke group in the current study had worse upper limb function than the cognitive impairment group, and this might be the reason. Although similar motor recovery of the upper and lower limbs following stroke, usually the upper limb has slower recovery rate compared to the lower limb^27^. On the contrary, the cognitive impairment group had more difficulty than the stroke group in using the telephone, transport, and managing finances, which require high cognitive demands. Using the telephone needs a series of actions to be performed, including picking up the phone, checking the number, making a call, and communicating with people. Transportation requires not only correct orientation but also driving or organizing other means of transport. Managing finances also requires more-complex thinking and organizational skills, such as paying bills and managing financial assets. Recent evidence showed that non-demented individuals with mild cognitive impairment (MCI) already have increasing difficulties with IADL tasks, particularly in using the telephone, transportation, taking medications, and handling their finances, which are more likely to be vulnerable to early cognitive decline^28, 29^. Nearly 60% of our participants in the cognitive impairment group had mild cognitive dysfunction, and therefore they had begun to have difficulty in the above-mentioned tasks. In fact, IADLs require greater interactions with physical and social environments and a greater degree of skill (e.g., problem-solving and social skills) than basic self-care tasks. Any underlying impairment in a person’s sensorimotor, cognitive-perceptual, or psychosocial capacity tends to affect the performance of IADL tasks to a greater degree than they affect basic self-care. Thus, individuals with cognitive impairment reasonably have more difficulties or need more assistance with IADL tasks.

It is also important to note that the most difficult BADL and IADL tasks were bathing and heavy housework respectively while the least difficult ones were eating and medication, regardless of the groups. It seems that some of the BADL or IADL tasks are inherently difficult or easy, and disorder only increases difficulty rather than changes the order of difficulty. Morris and colleagues reported a hierarchy of ADLs: the early loss function is hygiene, the mid-loss functions are toilet use and locomotion, and the late loss function is eating^30^, which is consistent with our findings. It was observed in clinical practice that bathing is the most difficult and least independent task in BADLs for disabled people because bathing involves a complex interplay of factors. Bathing can only be done in the bathroom, an environment that is relatively dangerous where people are most prone to falling. It may also require assistive devices and more procedures to complete the activity.

The difficulty of IADL tasks is more closely associated with cognitive demands. The more difficult IADLs require higher cognitive abilities. Loss of the ability to complete IADL tasks can be among the earliest signs of MCI, especially those cognitively demanding tasks^31^ or ones involving memory or complex reasoning, whereas more-basic activities might not yet be affected^32^. In the current study, only 30% of individuals in the cognitive impairment group were independent in BADLs and IADLs, but 60% of them had only mild cognitive dysfunction, indicating that cognitive function has great impacts on IADLs. In fact, a previous report showed that problems with IADLs could be indicative of an early cognitive disorder, and those with self-reported IADL impairment are more likely to convert to dementia^26^.

There are some limitations to this study. First, individuals in the present study were community-dwelling middle-aged and older adults, who may have had relatively better functional statuses and independent abilities with better cognitive and physical functions than individuals living in institutions. This limits the generalizability of our results to institution-living older populations and likely overestimated the disability measures. Second, for the cross-sectional design, because the exposure and outcome are simultaneously assessed, there is generally no evidence of a temporal relationship between exposure and outcome. In our study, therefore, we could not determine the causal relationship between the impairment and the disability. Third, because information was retrieved using questionnaires, the severity of the stroke and the post-stroke duration were unknown. Fourth, although this is a nationally representative sample with a sample size that exceeded 3000, the stroke group only accounted for 4%, the cognitive impairment group for 12%, and the combination group for 2% of the sample.

In summary, individuals with comorbidities of stroke and cognitive impairment had the highest prevalence of disabilities in both BADLs and IADLs, the greatest number of summed BADL and IADL tasks with disabilities, and the highest level of difficulty for each BADL and IADL task. However, similar prevalence, similar numbers of summed BADL and IADL tasks with disability, and similar levels of difficulty for each BADL and IADL task were found between the stroke group and cognitive impairment group, but the former had more difficult in dressing and the latter had more difficult in using the telephone, transport, and managing finances. For all participants, the most difficult BADL and IADL tasks were bathing and heavy housework respectively, while the least difficult ones were eating and taking medications. Our findings suggest that medical practitioners and clinical therapists should develop interventions or compensatory strategies, such as cognitive and physical training as well as ADL skill training, focus on secondary prevention of dementia and improve motor functional capacity to reduce loss of ADLs. Additionally, assessments and training of cognitive function after a stroke are important and necessary to reduce the severity of disabilities caused by the combined contributions from stroke and cognitive impairment.

## Methods

### Data source and study population

Data were obtained from the Taiwan Longitudinal Study on Aging (TLSA), Health and Welfare Data Science Center, Ministry of Health and Welfare (HWDC, MOHW), a nationwide representative survey conducted since 1989 with a follow-up every 3–4 years and a response rate up to 90%. The survey was initiated by the Taiwan Health Promotion Administration, Ministry of Health and Welfare as a collaborative effort with the University of Michigan. Details of the study design have been described previously^33–35^.The first interviews were conducted in 1989 (cohort I), and people aged 60 years and older living in the community were recruited. Follow-up surveys were conducted in 1993, 1996, 1999, 2003, 2007, and 2011. In 1996 (cohort II) and 2003 (cohort III), new samples of individuals aged 50 years and above were respectively interviewed. Death was the major cause of the decline in the sample size. Participants lost to attrition were more likely to have been older adults, males, and individuals who were socially inactive and had poorer physical functioning^36^. Data were collected through face-to-face interviews in Mandarin Chinese and included background information, household information, work history, and health status. The data used in this research were from the 2011 TLSA survey, which included the first, second, and third cohorts respectively enrolled in 1989, 1996, and 2003. The original sample comprised 3727 people, and those with incomplete demographic data, comorbidities, cognitive function, physical function, BADLs, or IADLs were excluded. The Taipei Medical University Joint Institutional Review Board (JIRB no. N201905090) approval and waivers for informed consent were obtained. This study was conducted in accordance with the Declaration of Helsinki.

Participants were categorized into four groups: control, stroke, cognitive impairment, and combination of stroke and cognitive impairment groups according to their history of stroke and scores on the Short Portable Mental State Questionnaire (SPMSQ)^37^, which has been commonly used in stroke survivors^38, 39^.A stroke diagnosis was either self- or proxy-reported affirmative to the question: “Has this disease been diagnosed by a doctor?” Cognitive impairment was identified based on the SPMSQ with more than three errors. Individuals without stroke or cognitive impairment served as a reference population (i.e., controls), while individuals with stroke and cognitive impairment comorbidity were classified into the combination group.

### Outcome measurements

#### Demographic data

Demographic characteristics were age (categorized as 55–65, 65–75, 75–85, and > 85 years), sex (male and female), marital status, educational level, comorbidities, cognitive function, and upper and lower limb functions. Marital status was categorized into married (married or living with a partner) and single (living without a partner such as separated, divorced, widowed, or never married). Educational level was determined by asking the participants how many years of formal education they had received and categorizing the answers into elementary school (including those who had received no formal education), junior high school, senior high school, and college and above. Self-reported comorbidities, including hypertension, diabetes, heart disease, cancer, lung disease, arthritis, and hyperlipidemia, were transformed into a comorbidity count classified as 0–2 and ≥ 3. Cognitive function was determined by the SPMSQ, which is a 10-item measure focusing on orientation, memory, and concentration. The Chinese version of SPMSQ has been validated with 0.70 for the test–retest reliability and 0.72 for the split-half coefficient of alpha^40, 41^. Scoring reflects the number of errors: 0–2 errors indicates normal mental functioning; 3 or 4 errors mild cognitive impairment; 5–7 errors moderate cognitive impairment, and ≥ 8 errors severe cognitive impairment. The internal consistency reliability of the SPMSQ in this study was 0.781. Upper limb function was defined by asking whether participants were able to hold or twist things with their fingers, while lower limb function was defined by asking whether participants were able to walk a distance of 200–300 m without the assistive device. The upper and lower limb functions used a 4-point Likert-scale in which a response of 0 indicated no difficulty and a response of 3 an inability to perform the function.

#### BADLs and IADLs

BADLs are commonly measured using the barthel index (BI)^42^, and various studies involving this index have been published in older adults^43^, patients with stroke^11^, and those with cognitive impairment^44^. Validity and reliability of Chinese version of BI have been substantiated in both Taiwanese and Chinese older adults^45, 46^. The current study used a six-item modified version of the original 10 items. Items included bathing, dressing, eating, transferring, indoor walking, and toileting. On the other hand, the Lawton-Brody Instrumental Activities of Daily Living Scale^8^ is a popular assessment of IADLs, which has been validated in a previous study^47^, and multiple studies involving this measurement have been published in older adults^48^, patients with stroke^49^, and those with cognitive impairment^50^. The current study used a nine-item modified version of the original eight items, with housework subdivided into light and heavy housework, and additionally including transport, managing finances, shopping, laundry, medication, food preparation, and using the telephone. Both BADLs and IADLs used a four-point Likert-scale with no difficulty (score of 0), some difficulty (1), great difficulty (2), and inability (3). Any item of BADLs or IADLs rated as having any degree of difficulty (> 0) was defined as a disability. For participants affected by cognitive impairment or illiteracy, a proxy respondent was used. The internal consistency reliabilities of the BADL and IADL measures in the present study were 0.930 and 0.921, respectively, determined by the Cronbach's alpha.

### Statistical analysis

Categorical variables are expressed as numbers and percentages, and continuous variables are expressed as means and standard deviations (SDs) without adjustments in the text, figures and tables. A Chi-squared test was used to identify differences in demographic factors, comorbidities, and cognitive and physical functions among the control, stroke, cognitive impairment, and combination groups. The same test was used again to compare prevalence of disabilities in BADLs and IADLs among the groups. In order to compare the numbers of disabled BADL and IADL tasks and the level of difficulty in each BADL and IADL task among groups, an analysis of covariance (ANCOVA) with demographics (sex, education, age, marital status, and number of comorbidities) as the covariates were implemented. ﻿In order to reduce the chance of a type I error, the more conservative Bonferroni post-hoc test was used. SPSS version 19 (SPSS, IBM, Armonk, NY, USA) was used, and the alpha level of statistical significance was set to 0.05.
